# Prevalence and risk factors for diastasis recti abdominis: a review and proposal of a new anatomical variation

**DOI:** 10.1007/s10029-021-02468-8

**Published:** 2021-08-06

**Authors:** M. Cavalli, A. Aiolfi, P. G. Bruni, L. Manfredini, F. Lombardo, M. T. Bonfanti, D. Bona, G. Campanelli

**Affiliations:** 1 Center of Research on the Pathology and High Specialization on the Abdominal Wall and Hernia Surgery, University of Insubria, Milano Hernia Center, Istituto Clinico Sant’Ambrogio, Via Faravelli 16, 20147 Milano, Italy; 2 Department of Biomedical Science for Health, Division of General Surgery, University of Milan, Istituto Clinico Sant’Ambrogio, Via Luigi Faravelli 16, 20147 Milan, Italy; 3 Casa Di Cura La Madonnina, via Quadronno 29, 20122 Milan, Italy

**Keywords:** Diastasis recti abdominis, Intra-rectus distance, Abdominal rectus muscle, Risk factor, Semilunar line

## Abstract

**Purpose:**

Diastasis recti abdominis (DRA) or rectus diastasis is an acquired condition in which the rectus muscles are separated by an abnormal distance along their length, but with no fascia defect.

To data there is no consensus about risk factors for DRA. The aim of this article is to critically review the literature about prevalence and risk factor of DRA.

**Method:**

A total of 13 papers were identified.

**Results:**

The real prevalence of DRA is unknown because the prevalence rate varies with measurement method, measurement site and judgment criteria, but it is certainly an extremely frequent condition. Numbers of parity, BMI, diabetes are the most plausible risk factors.

We identified a new anatomical variation in cadaveric dissection and in abdominal CT image evaluation: along the semilunar line the internal oblique aponeurosis could join the rectus sheath with only a posterior layer, so without a double layer (anterior and posterior) as usually described. We conducted a retrospective review of abdominal CT images and the presence of the posterior insertion only could be considered as a risk factor for DRA.

**Conclusion:**

Further studies with large sample size, including nulliparous, primiparous, pluriparous and men too, are necessary for identify the real prevalence

## Background

Diastasis recti abdominis (DRA) or rectus diastasis is an acquired condition in which the rectus muscles are separated by an abnormal distance along their length, but with no fascia defect. [1].

DRA is characterized by a protruding midline as a result of an increase in intra-abdominal pressure [2]. DRA involves a gradual thinning and widening of the linea alba, combined with a general laxity of the ventral abdominal wall muscle [3]. The musculofascial continuity of the midline and subsequent absence of a true hernia sac distinguishes DRA from a ventral hernia. However, thinning and stretching of the linea alba is an important risk factor for the actual development of midline hernias (umbilical, epigastric, trocar and incisional hernias) due to deterioration of the connective tissue and pulling of the abdominal muscles [4]. Even in a series of small umbilical and epigastric hernias (< 2 cm) concomitant rectus diastasis was diagnosed in 45% of patients [4].

The reported prevalence of DRA varies between studies and may be inaccurate due to different intra-rectus distance (IRD) cut-off values for the diagnosis, the use of different measurement assessment methods [5] (i.e., palpation vs. caliper vs. ultrasound vs computed tomography, rest vs. active) and measurement sites (single vs multiple, above, at or below the umbilicus).

DRA occurs most frequently during pregnancy and regresses spontaneously after childbirth in most women. However, at 12 months postpartum, 33% of women still experience DRA [6]. DRA has been found in 39% of older, parous women undergoing abdominal hysterectomy [7], and in 52% of urogynecological menopausal patients [8], suggesting that DRA can even persist past childbearing years. Data from nonparturient women are rare. Diastasis is also frequently present in men, but data regarding these cases are scarce.

To date, there is no consensus about risk factors for DRA. The aim of this article is to critically review the literature regarding the prevalence and risk factors of DRA, and to propose a new anatomical variation as a risk factor for DRA.

## Method

An extensive literature search was conducted by three authors (MC, AA and LM) to identify all English-written published articles on diastasis recti abdominis (DRA). PubMed, EMBASE, and Web of Science databases were consulted using the terms “DIASTASIS” and “RECTI” and “ABDOMINIS” and “INTRA-RECTUS” and “DISTANCE” until April 13th, 2021. The search was completed by consulting the listed references of each article [9]. Articles in non-English languages and those without a full available text were excluded.

All the articles, case reports, and case series were included in this narrative review, while abstracts were excluded [10]. Two authors (MC, AA) independently extracted data from eligible studies. Data extracted included study characteristics (first author name, year, and journal of publication), along with a number of patients included in the series, clinical and demographic characteristics of patients’ population, DRA evaluation, DRA definition, DRA prevalence and risk factors.

Informed consent was not necessary for the literature review.

## Results

A total of 13 papers were identified. Two articles were excluded because they were not in English, and five were excluded as the full text was unavailable (Table 1).

**Table 1:** List of paper evaluated

Author (year of the pubblication)	Type of study	Population		Type	DRA definition	DRA evaluation	Prevalence of DRA	Risk factor evaluated for DRA	Results
		N	Age						
Mota et al. (2015) [11]	Longitudinal observational study	84	32.1 ± 2.2	Primamaparus, at gestetional week 35, 6–8, 12–14 and 24–26 weeks PP	IRD > 16 mm	By US, 2 cm below the umbelicus	100% at gestetional week 35 52.4% at 6–8 week PP 53.6% at 12–14 weeks PP 39.3% at 6 months PP	Age, BMI before pregnancy BMI at 6 months post partum, weight gain during pregnancy, baby weight birth, abdominal circumference in late pregnancy, hypermobility, vaginal birth, regular exercise training	No risk factor for DRA at 6 months post-partum
Sperstad et al. (2016) [6]	Longitudinal observational study	117	28.7 ± 4.3	Nulliparous European women from 21 weeks gestation to 12 months PP	IRD > 2 fingerbreadths or observed protrusion along the linea alba	By palpation 4.5 cm above, at and 4.5 cm below the umbilicus in crunch position	33.1% at gestation week 21, 60% at 6 weeks PP 45.5% at 6 months PP 32.6% at 12 month PP	Age, height, mean weight before pregnancy, weight gain during pregnancy, delivery mode, baby’s birth weight, benign joint hypermobility syndrome, heavy lifting, level of abdominal and pelvic floor muscle exercise training 12 months PP, general exercise training 12 months PP	No risk factor for DRA at 12 months post partum
Spitznagle et al.(2007) [8]	Retrospective study	547	52.45 ± 16.65	Women presenting to urogynecological medical practice	A separation in rectus abdominis muscle	By palpation, 1in.above or below the umbilicus in stress position	52%, 35% in nulliparus	Age, race, parity, gravity, delivery type, menoapusal hormone replacement therapy, previous surgery	Risk factor for DRA: age, parity, gravity, caucasian or asian race, menopausal hormaone replacement therapy, previous abdominal surgery
Turan et al. (2011) [13]	Observative study	95	22.04 ± 1.29	Nulliparus, primiparou and multiparous	IRD > 2 cm	By palpation, 3–4 cm above the umbilicus	0% in nulliparous, 2% in primiparous 59% in pluriparous	Parity type of delivery	Risk factor for DRA: parity, delivery type for those that gave birth twice
Wu (2020) [14]	Observative study	644	58	Women who had received a CT scan	< 45 years: IRD > 1.0 cm above the umbilicus, 2.7 cm at the periumbilicus, 0.9 cm below the umbilicus > 45 years: IRD > 1.5 cm above the umbilicus, 2.7 cm at the umbilicus, 1.4 cm below the umbilicus	By CT	28.40%	Age, BMI, chronich cough, chronic constipation, diabetes, parity	Risk factor for DRA: BMI, diabetes, parity
McPhail [16]	Observative study	42	73.2 in AAA group 70.8 in PAD group	Men referring to a vascular practice for AAA or PAD	A visible midline bulge through the linea above the umbilicus in stress position	Phisical examination	66.7% in AAA group 16.7% in PAD group	NA	Prevalence of DRA is different between the two group
Gitta et al. [32]	Article in Hungarian								
Rett et al. [33]	Article in Portuguese								
Wang et al. [35]	Full text NA								
Candido et al. [34]	Full text NA								
Champion [37]	Full text NA								
Doubkova [36]	Full text NA								
Zhou [38]	Full text NA								

*IRD* inter recti distance, *DRA* diastasis rectus abdominis, *NA* not available, *AAA* abdominal aortuc aneu;rysm, *PAD* peripheral arterial occlusive disease, *BMI* body mass index, *CT* computer tomography, *US* ultrasound, *PP* post partum

Mota et al. [11] investigated possible risk factors for primiparous women with and without DRA at 6 months postpartum (sample size 84 women). The cut-off value for DRA was set at intra-rectus distance (IRD) > 16 mm by ultrasound measurement at 2 cm below the umbilicus, using the definition outlined by Beer et al. [12]. Risk factors evaluated were age, BMI before pregnancy and at 6 months postpartum, weight gain during pregnancy, Beighton’s hypermobility score, baby weight at birth, abdominal circumference at gestational week 35, exercise training level before, during and after pregnancy, and type of delivery. There were no statistically significant differences between groups for any factor. Prevalence of DRA was 39.3% 6 months post-partum. Limitations of this study were the lack of pre-pregnancy assessment of inter-recti distances, the measurement at just one point [2 cm below the umbilicus] and the inclusion of only primiparous women.

Sperstad et al. [6] conducted a longitudinal study following a cohort of 117 nulliparous European women from 21 weeks gestation to 12 months postpartum. DRA was measured by palpation at the umbilicus, as well as 4.5 cm above and 4.5 cm below the umbilicus in the crunch position. DRA was set as a separation of > 2 fingerbreadths or observed protrusion along the linea alba. Risk factors that were assessed included age, height, mean weight before this pregnancy, weight gain during pregnancy, delivery mode, baby’s birth weight, benign joint hypermobility syndrome (BJHS) assessed with Beighton score, heavy lifting, and level of abdominal and pelvic floor muscle exercise training and general exercise training at 12 months postpartum. The study found no significant differences in evaluated risk factors when comparing women with and without DRA at 12 months postpartum. Prevalence of DRA was 32.6% 12 months post-partum, and the majority had mild DRA (only two women had moderate DRA and none had severe DRA). The limitations of this study were again the lack of data on the normal width of linea alba before pregnancy and the inclusion of only nulliparous women carrying a single fetus. Moreover, the manual evaluation of DRA could be an important bias. The author concluded that the fact that the majority of women had mild DRA could be a reason for not seeing any differences between the groups regarding all the risk factors.

Spitznagle et al. [8] proposed a retrospective study of the medical charts from a cohort of 547 patients who presented to a urogynecological medical practice. DRA was defined as a separation in the rectus abdominis muscle 1 in. above or below the umbilicus. The overall prevalence of DRA was 52%, prevalence in nulliparous was 35%. The patients with DRA were older and reported higher parity and gravity levels. Compared to the patients without DRA, a larger percentage of patients with DRA were Caucasian or Asian, menopausal, using hormone replacement therapy, and had surgery in the abdominal region. Limitations of this study are that it is a retrospective chart review and that DRA was evaluated by palpation, but this large series of patients reported—for the first time—DRA impairment in nulliparous women, and suggested that DRA can persist beyond childbearing years.

Turan et al. [13] proposed an observative study in 95 young women (nulliparous, primiparous and multiparous) referring to the gynecology practice for vaginal discharge and examined for the presence of DRA. Any separation between the medial edges of the rectus muscles that was greater than 2 cm width by palpation, in a stress position, constituted a diagnosis of DRA. DRA prevalence was 24%, and a positive correlation was established between parity and DRA; DRA was not detected in the nulliparous group (in contrast to results by Spitznagle); but DRA was present in 2% of primiparous and 59% of multiparous patients. Comparing patients in terms of delivery type resulted in an insignificant result for patients that had given birth once; however, the difference was significant for patients that had given birth twice, with a larger IRD for women who had had a cesarean delivery. A limitation of this study was the manual evaluation of the IRD.

Wu [14] presented results from a survey of 644 adult women who had received an abdominal computed tomography (CT) scan. Patients who had had previous abdominal surgery and/or a pregnancy or postpartum within 1 year were excluded. The distance of the inter-recti regions was measured at the umbilicus, and at 4.5 cm above and 4.5 cm below the umbilicus, by means of CT images. According to the Rath classification [15], DRA was defined as a separation of the two recti more than 1.0 cm above the umbilicus, 2.7 cm at the peri-umbilicus and 0.9 cm below the umbilicus for subjects younger than 45 years; for subjects over 45 years, the corresponding values were 1.5 cm, 2.7 cm and 1.4 cm, respectively. The overall prevalence of DRA was 28.,4%, but after age stratification, DRA was present in 50.9% of young people (< 45), 24.6% of middle-aged subjects (45–59) and 22.7% of elderly people (≥ 60). Therefore, the risk of DRA decreased with age (in contrast to results by Spitzagle) but increased with the number of pregnancies (as previously reported by Spitznagle and Turan). This study found that BMI and diabetes are influential factors for the occurrence of DRA. The strength of this study is the large series of women (nulliparous, primiparous and multiparous) included, and the use of CT for DRA evaluation and measurement.

Just one study has presented data regarding DRA in men: McPhail [16] reported a small sample series of men (42) who had referred to a vascular practice for the evaluation of abdominal aortic aneurysm (AAA) and peripheral arterial occlusive disease (PAD). DRA was defined as a visible midline bulge through the linea above the umbilicus in a stress position. Diastasis recti were present in 66.7% of patients with AAA versus 16.7% of patients with PAD. Limitations of this study were the small sample size and the subjective grading of the presence or absence of diastasis recti.

## Discussion

### Data about DRA are too scarce and heterogeneous for a systematic review

The need for deeper clarity and uniformity in DRA has been well understood by the German Hernia Society (DHG) and the International Endohernia Society (IEHS). They, therefore, proposed a classification for DRA [1]. Nevertheless, strong indications about the definition and evaluation of DRA are lacking.

The prevalence rate of DRA varies with measurement method, measurement site and judgment criteria. The finger-width method is widely used, as it is relatively convenient and economical compared with other methods. However, due to individual differences in finger width, the accuracy of outcome is not reliable. In addition, certain conditions, such as thick subcutaneous fat and significant abdominal slack, can make diagnosis difficult with this technique. Using ultrasound or CT to measure IRD is more accurate than using finger width.

Moreover, the lack of a uniform definition and classification is a barrier to comparing outcomes.

Most researchers focused on pregnant women and postpartum women but paid little attention to women more than 1 year postpartum or middle-aged and elderly women.

No significant differences were found for age as a risk factor, by Mota and Sperstad, but they only included primiparous women in their studies, so this was a series with a small age span. Conversely, age is considered a risk factor by Spitznagle, but a protective factor by Wu et al., who explained the high incidence of DRA in young women because pregnancies are mostly concentrated during this period, but women with a pregnancy or postpartum within 1 year were excluded in the study by Wu, and the IRD tends to become significantly smaller during the first 12 months postpartum, and the greatest recovery tends to occur between day 1 and 8 weeks after delivery when the IRD reached a plateau [17].

Pregnancy is confirmed to be a risk factor for DRA and, moreover, the more pregnancies a woman has had, the more likely she is to develop DRA. Cesarian section only seems to be a risk factor for women who have given birth twice. The viscoelastic properties inherent to the collagen make the linea alba prone to increasing length when the mechanical stress is prolonged in time, as in the case of lasting increased intra-abdominal pressure [18]. Long-lasting increased intra-abdominal pressure from a growing fetus and expanding uterus combined with hormonal changes (increasing in secretion of relaxin, progesterone and estrogen) on connective tissue create a physiological (normal) widening of the IRD, creating a DRA during pregnancy. The anterolateral abdominal wall undergoes dramatic changes as the pregnancy progresses. The two muscle bellies of the rectus abdominis elongate and curve round as the abdominal wall expands, similar to suspenders on obese men. At 38 weeks gestation, the length of the abdominal muscles increases to a mean of 115% compared to the beginning of pregnancy. The infra-umbilical (from umbilicus to symphysis pubis) region of the linea alba has a greater number of transverse fibers, which provides a greater ability to resist the tensile stresses imposed thereon [19, 20]. Liaw et al. [21] noted that, during pregnancy, the infraumbilical region could sustain a longer duration of stretch during pregnancy (as the growing uterus rises out of the pelvis at 12 weeks and makes contact with the abdominal wall). Their data indicated that IRD values were larger for locations above the umbilicus compared to those below the umbilicus, and suggested that the infraumbilical region of the linea alba has a greater ability to resist stresses imposed over a longer period of time [21].

According to Wu et al., BMI is an influential factor for the occurrence of DRA. The possible reason is that obese people usually have more adipose tissue in the abdominal cavity, such as greater omentum and mesentery, resulting in an increase in abdominal contents and pressure on the abdominal wall, which—in turn—causes the separation of rectus abdominis to both sides. In addition, obesity may occur at the same time as muscle loss [22]. Moreover, similar results from the study proposed by Grossi et al. showed that the amount of collagen in the linea alba above the umbilical region in morbidly obese patients is smaller than in non-obese cadavers of the same age group [23]. Therefore, all these factors probably result in the occurrence of DRA in obese patients.

Moreover, Wu indicated that DRA was related to diabetes. Diabetes can cause loss of muscle mass and function, and cause sarcopenia [24, 25]. The changes in the rectus abdominis caused by diabetes may have the following two mechanisms: diabetes could contribute to impaired mitochondrial oxidative phosphorylation and hypercomplex assembly in rectus abdominis muscle fibers [26]. It could also induce muscle structure change by reducing fast fibers and increasing slow fibers [27].

The high incidence of diastasis recti in subjects with AAA suggests an underlying weakness of connective tissue. Smoking causes an acquired weakness of connective tissue, and it has also been associated with an increased risk of incisional and recurrent groin hernia [28, 29]. Hydroxyproline, a compound early in the collagen synthesis pathway, has been described as constituting 80% of the dry weight of the rectus sheath, with lower levels in those with inguinal hernias [30]. Smokers have been shown to produce less hydroxyproline than nonsmokers [31]. These findings could explain weaknesses in both the linea alba and the aortic wall.

With the aim of exploring the anatomical interactions between the rectus muscle and lateral muscles (external oblique, internal oblique and transverse abdominal muscles) and of understanding the real etiopathogenesis of DRA, we carried out some cadaveric dissections. During these anatomical evaluations, we noted that the internal oblique aponeurosis can join the rectus sheath in two ways, namely (a) splitting its fibers in an anterior and posterior layer, as classically described, or (b) joining only the posterior rectus sheath without an anterior layer.

As usually described, at the lateral margin of the rectus sheath, the lateral muscles aponeurosis join themselves in the semilunar or spigelius line. The external oblique aponeurosis constantly passes in front of the rectus muscles, composing the anterior lamina of the sheath. The internal oblique aponeurosis splits its fibers in an anterior and a posterior layer. The anterior layer joins the fibers of the external oblique in front of the rectus muscle to constitute the anterior lamina. However, some centimeters below the umbilicus, no split in the fibers is evident, and all the aponeurosis of the internal oblique join the external oblique and transverse aponeurosis in constituting the anterior sheath. The transverse muscle aponeurosis also behaves differently at a cranial level compared to the caudal level. Cranially, the fibers constantly remain posterior to the rectus and constitute the deep layer of the sheath, but—at a variable level—some centimeters below the umbilicus, they present anteriorly with all other flat muscle aponeurosis.

To confirm our new variation in the semilunar line, we conducted a retrospective review of abdominal CT images. A blinded trained radiologist evaluated the CT abdominal images of 100 patients (men and women), randomly selected from patients who had been referred to the Radiology Service for any pathological indication (abdominal wall pathology, vascular pathology, urological pathology, gastrointestinal pathology). In compliance with privacy laws, no information about personal details was available.

In 89/100 (89%) patients, the classic insertion (anterior and posterior) was present, but in 11/100 (11%) patients, only a posterior insertion was present. In patients presenting the classical insertion, only 23/89 (26%) CT images showed a rectus diastasis (according to the Rath definition [15]), whilst in patients with only the posterior insertion, diastasis was present in all patients (100%).

It seems that only the posterior insertion could be considered as a risk factor for diastasis.

A descriptive observational cross-sectional prospective study with a large sample size will soon begin, with the aim of confirming our hypothesis.

## Conclusions

The actual prevalence of DRA is unknown because the prevalence rate varies according to the measurement method, measurement site and judgment criteria, but it is certainly an extremely frequent condition.

It is equally uncertain whether DRA is to be considered a pathological condition or a natural part of aging, and the risk factor for DRA have not been clearly defined. Numbers of parity, BMI, and diabetes are the most plausible risk factors, but further studies with large sample sizes, including nulliparous, primiparous, and pluriparous women, as well as men, are necessary.

A deeper knowledge of diastasis and its risk factors, and a greater understanding of the anatomy of the semilunar line could offer the possibility of identifying groups of patients at risk for developing diastasis and abdominal wall pathologies (primary ventral hernia and incisional hernia), to develop new surgical techniques and implement and improve existing ones.
